# Impact of operating room technology on intra-operative nurses' workload and job satisfaction: An observational study

**DOI:** 10.1016/j.ijnsa.2025.100341

**Published:** 2025-04-29

**Authors:** Anne M. Schouten, Rick M. Butler, Carlijn E. Vrins, Steven M. Flipse, Frank Willem Jansen, Anne C. van der Eijk, John J. van den Dobbelsteen

**Affiliations:** a Technical University of Delft, Biomedical Engineering department, Mekelweg 5, 2628 CD, Delft, the Netherlands; b Technical University of Delft, Science Education and Communication department, Mekelweg 5, 2628 CD, Delft, the Netherlands; c Leiden University Medical Center, Gynecology department, Albinusdreef 2, 2333 ZA, Leiden, the Netherlands; d Leiden University Medical Center, Operation Room Centre, Albinusdreef 2, 2333 ZA, Leiden, the Netherlands

**Keywords:** Operating room technology, Intra-operative nurses, Robotic surgery, Healthcare workforce, Workload, Job satisfaction

## Abstract

**Background:**

The integration of medical technology in the operating room has revolutionized surgical workflows and team dynamics. However, this progress coincides with a critical global shortage of nurses and a high turnover rate within the existing nursing workforce, impacting patient care quality, nurses' well-being, and hospital finances

**Aim:**

This study investigates the impact of technological complexity on the workload and job satisfaction of intra-operative nurses, focusing on open surgery, minimally invasive surgery, and robotic-assisted surgery within the gynecology department of a Dutch academic hospital.

**Method:**

The study design follows a mixed-methods approach, combining qualitative and quantitative methods to assess nursing experiences across three surgical modalities. Specifically, we conducted 5 interviews, distributed 28 validated questionnaires, performed automated video analysis on 35 recorded surgeries, and analyzed hospital datasets encompassing 411 cases. Data collection took place in 2022 and 2023.

**Results:**

Findings show that intra-operative nurses experience varying levels of workload and job satisfaction depending on the level of technology. Open procedures showed the highest job satisfaction, characterized by continuous engagement and manageable workloads. Minimally invasive surgery procedures, while less physically demanding, were associated with reduced involvement and lower satisfaction. Robotic-assisted procedures presented the most significant challenges, with increased workload, reduced involvement, and heightened stress stemming from surgery preparation, technological complexity, and altered team dynamics.

**Conclusions:**

Advancements in medical technology improve outcomes and efficiency but often neglect their impact on intra-operative nurses. Communication issues, equipment challenges, and limited technical training contribute to burnout and turnover. This study underscores the need for supportive operating room environments that prioritize nurses’ well-being. By examining the link between technology, workload, and satisfaction, it offers strategies to retain and empower nursing staff. It also shows how automated video analysis can objectively assess nursing roles, highlighting the importance of balancing technology with human-centered care in the operating room.

**Study registration:**

Not registered

What is already known.

• Intra-operative nurses face unique challenges in technologically advanced surgical environments.
• Different surgical modalities require varying skill sets, team dynamics, and levels of nurse involvement.
• Increased technology in the operating room can heighten stress and workload for surgical teams.

What this paper adds.

• Open surgery provides the highest job satisfaction, while robotic-assisted surgery increases the experienced workload and reduces nurse engagement.
• Automated video analysis offers a novel method to objectively quantify nurses’ roles across surgical technologies.
• Supportive operating room environments should be taken into account when address nursing shortages and foster job satisfaction in high-tech settings.

## 1 Introduction

Technology has become indispensable in the operating room (Zhang et al., 2021). The advancement of medical technology is illustrated by the shift from traditional surgical methods, open surgery and minimally invasive surgery, to robotic-assisted surgery (Sheetz et al., 2020). When comparing robotic-assisted surgery to open surgery and minimally invasive surgery, literature shows that robotic-assisted surgery typically takes longer, requires larger teams, and involves more complex equipment. Likewise, open surgery and minimally invasive surgery each require distinct skills and team dynamics (Zheng et al., 2015). Consequently, each type of surgery functions within a unique working environment and demands specific skill sets (Gjeraa et al., 2016).

The surge in medical technology brings advantages: the primary goals when developing medical technology for the operating room are to enhance patient outcomes, increase time efficiency, and improve surgeons' well-being (Schouten et al., 2023). However, the adoption of technology has also introduced new challenges for the surgical team as it increases the complexity of procedural workflows. Several papers address the impact of the use of technology on team composition and communication, as well as the influence of experiences of individual operating room team members (L Cao et al., 2004; Webster & Cao, 2006). For example, certain aspects of the altered working environment, such as longer surgery durations and more intricate procedures, are associated with heightened stress and workload for the surgical team. (Gillespie et al., 2021).

Despite significant changes in their work, there is a paucity in literature on the effects of technology on the work of nurses (Schuessler et al., 2020). Meanwhile, there is a growing concern that the nursing profession is facing labour shortages due to a high turnover and inequitable workforce distribution. The causes related to the nursing shortage are numerous and impacting patient care quality, nurses' well-being, and hospital finances (Lee et al., 2020). In particular, the recruitment and retention of intra-operative nurses are challenging, due to the need to collaborate with various health professionals in a fast-paced, high-tech environment with high patient turnover (Björn et al., 2015). Compared to other nursing specialties, intra-operative nurses report less favorable working conditions, such as limitations of communication in the team, feeling of isolation, blocking of vision due to large equipment, unexpected device errors and malfunctions, fear and anxiety due to lack of technical knowledge and burnout due to lack of trained nurses (Senol Celik et al., 2023).

Smith and Palesy (2018) introduced the concept of *“technology stress”* in perioperative nursing – the idea that the influx of advanced technology, while improving certain tasks, can also create stress as nurses strive to master new devices and systems​. They argue that in many hospitals, perioperative nurses often become “super users” of new operating room technologies, rapidly learning the equipment and then training their colleagues​. This expanded role means that nurses not only deliver patient care but also serve as on-the-spot technical experts and troubleshooters for complex machines. Smith and Palesy (2018) have noted an ongoing tension between *technical* and *caring* aspects of nursing practice in the OR, as nurses balance operating sophisticated devices with their traditional patient-focused duties​.

To enhance and maintain a robust intra-operative nursing workforce, it is necessary to cultivate a work environment that inspires and motivates nurses (Lambrou et al., 2010). A supportive and engaging atmosphere encourages job satisfaction and enhances overall team performance. This not only ensures that nurses feel valued and appreciated but also strengthens their commitment to delivering high-quality care during surgical procedures (Gusar et al., 2020). It is therefore important to understand the relationships between the characteristics of their working environment, job satisfaction, and workload (Lee et al., 2020). Most perioperative nursing research, however, does not differientiate between levels of technological complexity in operating room environments (Kelvered et al., 2012; Sørensen et al., 2014). Literature highlights the need for comparative studies between high-tech and low-tech operating rooms to, for example, better understand how nurses allocate attention between patients and technology (Bull and FitzGerald, 2006; Göras et al., 2019).

In this study, we investigate the impact of medical technology on the workload and job satisfaction of intra-operative nurses by comparing these factors across open surgery, minimally invasive surgery, and robotic-assisted surgery. The study design follows a mixed-methods approach, combining a qualitative analysis of interviews with a focused quantitative analysis using questionnaires, video recordings, and hospital data (Fig. 2).

By conducting interviews and administering validated questionnaires we obtain insight in how nurses perceive and experience the working conditions in surgical environments with different technological complexity. We hypothesize that nurses prioritize the physical and emotional aspects of patient care above a focus on handling medical technology and that such interactions positively influence their job satisfaction. Next to this qualitative research approach, we explore the potential of automated video analysis to quantify the role and tasks of intra-operative nurses across the afore mentioned types of surgery. Automated video analysis offers key advantages over traditional methods like direct observation or self-reports. It reduces recall and observer bias, minimizes disruption in the operating room, and provides objective, consistent, and detailed data. This allows for more accurate comparisons across surgery types and enables retrospective analysis to validate findings from other sources (Gabriel et al., 2024).

## 2 Method

**Fig. 1:**
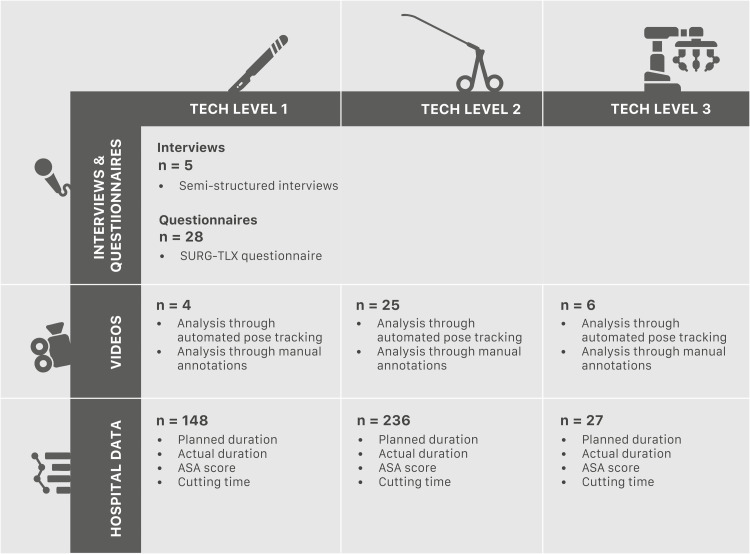
Analysis of three levels of surgical technology within the gynecology department at LUMC, based on three data sources: interviews and questionnaires with nurses experienced in all three technology levels, video recordings, and hospital data from 2023.

At the Leiden University Centre (LUMC) in the Netherlands, we collected three types of data within the gynaecology department in 2022 and 2023: interviews and questionnaires with intra-operative nurses, video recordings of gynecology surgeries, and hospital datasets of gynecology surgeries. The surgeries were categorized into three levels of technology: open surgery as the lowest level, minimally invasive surgery as the second level, robotic-assisted surgery as the highest level. Fig. 1 gives an overview of the collected data used for the analysis of the three technology levels.

In the study, five surgical approaches used in gynecology were incorporated: (1) open surgery, (2) vaginal surgery, (3) hysteroscopic surgery, (4) laparoscopic surgery, and (5) robotic-assisted surgery. These approaches were distributed across the three technology levels as follows:

• *Tech level 1:* open surgery- Open surgical procedures.
• *Tech level 2:* minimally invasive surgery - Includes vaginal surgery, hysteroscopic surgery, and laparoscopic surgery.
• *Tech level 3:* robotic-assisted surgery - Procedures performed with robotic assistance.

### 2.1 Study design

**Fig. 2:**
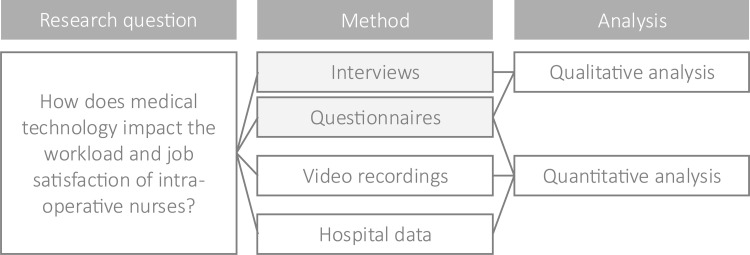
Flowchart with study design.

The study design follows a mixed-methods approach, integrating qualitative interviews with a focused quantitative analysis based on questionnaires, video recordings, and hospital data (Fig. 2). The questionnaire consisted of two parts: the first part was analyzed qualitatively to identify factors influencing workload and job satisfaction, while the second part—based on the Surgery Task Load Index (Wilson et al., 2011)—was analyzed quantitatively to compare perceived workload and satisfaction across different surgery types and phases.

### 2.2 Interviews and questionnaires

We conducted interviews and questionnaires based on the validated survey structure of Surgery Task Load Index (Wilson et al., 2011) for each type of surgery (open surgery, minimally invasive surgery and robotic-assisted surgery), to identify factors that influence workload and job satisfaction. Participants included intra-operative nurses with experience in all three types of surgery. Five nurses participated in semi-structured interviews, while 28 completed a paper-based questionnaire. Of these, 19 nurses fully completed the Surgery Task Load Index section. Participation was voluntary. For the questionnaire, one of the researchers was present in the operating room cafeteria, and nurses filled in the form when they had time. The questionnaire was filled out anonymously.

First, intra-operative nurses completed the questionnaire individually. To gain a better impression of overall practices, they were asked to reflect on their general experiences with the three types of surgery, rather than on a specific procedure they had recently assisted in. This approach also encouraged a broader range of responses and helped increase the response rate. In the first section of the questionnaire, we gathered general information about the participants, such as their age, years of experience, and specialty. An open-ended question asked participants to identify at least three factors that contribute to their job satisfaction and three factors that influence their perceived workload. In the second section, participants were required to complete a Surgery Task Load Index based survey, developed for this study.

The original Surgery Task Load Index evaluates six aspects on a scale of 20: *mental demands, physical demands, temporal demands, task complexity, situational stress* and *distractions.* To capture both workload and job satisfaction, we added a seventh aspect, *job satisfaction*, to the Surgery Task Load Index. This is, to our knowledge, the first time that job satisfaction has been integrated into the Surgery Task Load Index framework. As such, this represents an initial construct and an exploratory application of the tool. The job satisfaction aspect was measured using the same 20-point scale to assess how nurses perceive their satisfaction during surgeries, enabling us to explore the potential relationship between workload and job satisfaction in the operating room. Given the novelty of this addition, the outcomes related to job satisfaction should be interpreted as indicative rather than definitive—providing direction for future research rather than serving as hard statistical conclusions.

To facilitate comparisons among open surgery, minimally invasive surgery, and robotic-assisted surgery for each aspect, we added a separate scale for each surgery type (see Supplementary Fig. 1 in Appendix A). All aspects are scored this way for three clinical phases (corresponding with the registered timestaps of the hospital) of the surgery: (Phase 1) patient entry to first incision; (Phase 2) first incision to closing; (Phase 3) closing to patient exit.

In individual semi-structured interviews with five intra-operative nurses, we delved deeper into the questionnaire questions to gain a richer understanding of their perspectives. Interviews were conducted following email invitations, with participants selected based on recommendations from the staff team leader. Participants were asked to explain the context behind the factors they considered important for their job satisfaction and workload. The interview responses were transcribed using WisperAI (v20231117) (WisperAI Inc., 2023). To analyze the data, we used inductive categorization, a method where patterns and themes emerge directly from the data without relying on pre-existing frameworks. This approach allowed us to group the factors identified by participants into overarching themes that reflect their insights into workload and job satisfaction.

### 2.3 Video analysis

To quantify the factors identified in the interviews and questionnaires, we analyzed video recordings of surgeries. Over the course of a year, gynecological surgeries were filmed in two ORs using multiple cameras mounted on the ceiling. The videos were collected on computers running Noldus Observer XT software (Noldus Information Technology, 2023). The research setup consisted of the operating room with two Axis wide-view cameras mounted on the ceiling. These cameras were connected via ceiling-mounted cables to the recording computer in an adjacent room. The second operating room had four cameras installed in a similar manner, connected to a second recording computer. Before the filming process began, the study setup was ethically approved by the scientific review committee of the Leiden Univeristy Medical Center. Procedures were filmed only if all members of the surgical team consented to participate and if the patient had signed a consent form at least 24 hours in advance. To ensure privacy, the faces of the staff and the entire body of the patient were blurred. The videos were stored and analyzed in the Leiden Univeristy Medical Center, according to the data compliance plan in Appendix B. Surgeries that deviated from the planned procedure, such as those impacted by complications, were excluded from the study.

#### 2.3.1 Manual annotations

To estimate the frequency of critical factors influencing work satisfaction and workload, we selected the same camera viewpoint for all videos and manually annotated the actions of nurses, the overall operating room team, and the room conditions using Noldus software. The annotation scheme used is detailed in Supplementary Table 1, Appendix C. The annotations in Noldus were exported to Microsoft Excel (Microsoft Corporation, 2023) for quantitative data analysis.

#### 2.3.2 Automated pose tracking

We used automated human-pose detection to extract movements and locations of the staff during surgery. Detection of staff in the videos was accomplished with AlphaPose (Fang et al., 2023), which is an open-source framework for human pose estimation that uses deep learning models. It estimates human poses by identifying the positions of joints such as shoulders, elbows, and knees in images or videos.

**Fig. 3:**
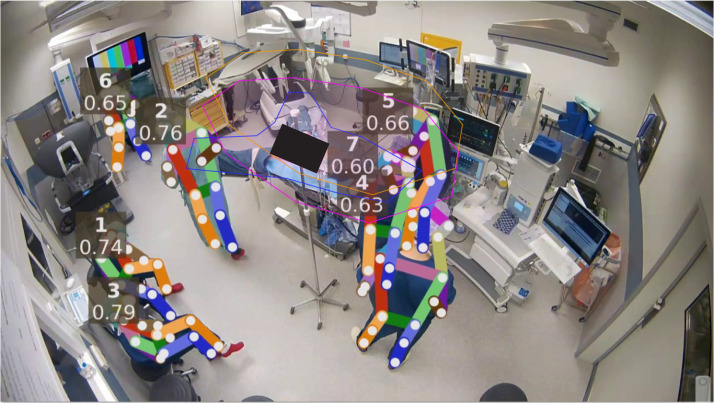
Frame of one of the recorded videos in which the poses of the staff (1-7) and the operation table area have been annotated. If the hands of a staff member are in the blue-line marked area, the shoulders in the pink area and the head in the orange area, it was counted as "active at the operation table”.

Specifically, AlphaPose was used to detect staff poses in individual video frames. A version of BYTE (*Better Yet Tracking by Exploiting the Overlap*) (Butler et al., 2025), adapted to leverage pose data, was employed to track individual staff members between video frames. To quantify the time staff spends actively at the operating table, we monitored the presence of three specific pose subsets in designated annotated areas, as shown in Fig. 3. This metric was chosen because we hypothesized that the time spent actively at the operating table could serve as an indicator of the nurse’s active involvement during surgery. To identify this involvement, we required both wrists to be near the patient, both shoulders to be positioned above the wrists, and the head (defined as a subset of ears, eyes, and nose) to be in a third area above the shoulders. These latter two pose subsets were included to mitigate the impact of camera perspective, ensuring that a wrist from someone not actively working at the operating table would not be misclassified as involvement.

In addition to positional constraints, movement thresholds were implemented to prevent false positives from staff merely passing by the operating table without interacting with it. Movement speed was measured in pixels by analyzing the positions of key points in the poses across consecutive frames. If the movement of the shoulders and head exceeded a threshold of 17.5 pixels over 5 frames, the individual was classified as not being active at the operating table. Wrist motion was not taken in account, as the wrists are expected to move during activity. Also, leg motion was not computed as it is difficult to detect them accurately in this setting due to the wearing of surgical aprons and camera angles. Data analysis was performed using R (R Core Team, 2023).

### 2.4 Hospital data

To provide an objective context for the interview and questionnaire findings, we analyzed hospital data from 686 gynecology procedures conducted between January 2, 2023, and December 29, 2023. The dataset included details such as staff composition, procedure indications, patient risk scores (ASA Physical Status Classification System), emergency classifications, and the planned duration of each procedure phase, as recorded in the hospital information system HIX 6.3 (Chipsoft, 2023). This analysis aimed to quantify how often the factors mentioned by nurses in the interviews and questionnaires occurred.

### 2.5 Handling of missing data

Missing values were handled as follows: for the Surgery Task Load Index questionnaire, only fully completed responses were included in the quantitative analysis (*n* = 19). Partially completed questionnaires were excluded from the analysis of that specific section but included in the qualitative analysis of the open-ended questions, where applicable. No imputation techniques were used. For the hospital data and video recordings, only complete cases with full information were included in the corresponding analyses.

### 2.6 Ethics

Approval for this research was obtained from the Human Research Ethics Committee (HREC) on March 25, 2024, under application number 3822.

### 2.7 Statistical analysis

For the SURG-TLX outcomes, a non-parametric Friedman test was performed on the data to assess whether there were statistically significant differences in the importance of the SURGTLX domains. If the Friedman test indicated a statistically significant difference (p < 0.05), post-hoc analysis using the Nemenyi test was conducted to identify which specific domains differed from each other. Effect sizes were reported using partial eta squared (η²) to indicate the strength of associations. Statistical analyses were conducted using IBM SPSS Statistics (Version 29.0.0.0 (241)) (IBM SPSS Statistics, 2023) to determine the statistical significance of the findings.

## 3 Results

This section begins with the presentation of the interview and questionnaire results, followed by the findings from the automated pose detection analysis of the video data. Finally, the results of the hospital data analysis are presented.

### 3.1 Interviews and questionnaire results

We conducted five semi-structured interviews and administered 28 questionnaires. The demographic characteristics of the participants are shown in Table 1.

**Table 1:** Demographic characteristics of the participants.

**Characteristic**	**Mean**	**SD**	**Min**	**Max**
Number of women	26 (93%)	-	-	-
Age (years)	36.4	5.2	25	50
Total work experience (years)	14.18	9.92	1	40
Experience with robotic surgery (years)	4.19	2.76	0	10
Working hours per week	30.71	4.30	24	36

**Fig. 4:**
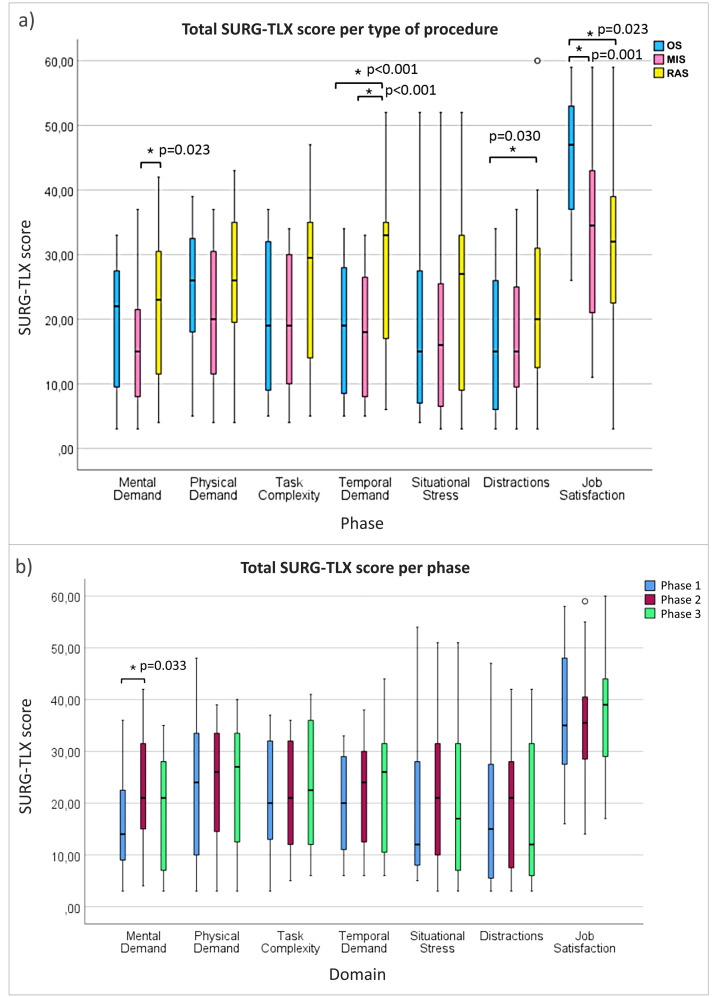
a) Boxplot illustrating the SURG-TLX scores for each surgical procedure type: open surgery (OS), minimally invasive surgery (MIS), and robot assisited surgery (RAS). The scores are derived by summing the scores for each procedure type across all surgical phases (Phase 1, Phase 2, Phase 3). This figure emphasizes the variations in perceived workload and job satisfaction across the three procedure types, with * indicating statistically significant differences. b) Boxplot illustrating the SURG-TLX scores for each surgical phase (Phase 1, Phase 2, and Phase 3). The scores represent the sum of individual scores across all procedure types.

The Surgery Task Load Index results showed significant differences in mental demand, temporal demand, distractions, and job satisfaction across procedure types (Fig. 4a). Robotic-assisted surgery was associated with higher perceived mental and temporal demand compared to minimally invasive and open procedures, while open surgery was linked to the highest job satisfaction. Full descriptive statistics and effect sizes are provided in Table 2.

**Table 2:** Descriptive statistics and effect sizes for SURG-TLX domains with significant differences by procedure type (open surgery OS, minimally invasive surgery MIS and robotic-assisted surgery RAS).

**Domain**	**Procedure type**	**Mean (SD)**	**Significat differences**	**Effect size (partial η²)**
Mental demand	Open	18.95 (10.96)	Robot > Minimally invasive (p = 0.023)	∼0.06 (small–moderate)
	Minimally invasive	16.00 (10.42)		
	Robot	22.58 (11.41)		
Temporal demand	Open	20.26 (11.39)	Robot > Open, Minimally invasive (p < 0.001)	∼0.09 (moderate)
	Minimally invasive	19.68 (10.27)		
	Robot	25.37 (12.43)		
Distractions	Open	16.16 (10.87)	Robot > Open (p = 0.030)	∼0.07 (moderate)
	Minimally invasive	16.84 (10.23)		
	Robot	23.05 (14.40)		
Job satisfaction	Open	46.47 (8.53)	Open > Minimally invasive (p = 0.023), Open > Robot (p = 0.001)	∼0.12 (moderate–large)
	Minimally invasive	34.53 (13.83)		
	Robot	31.95 (14.27)		

In terms of overall workload, mean Surgery Task Load Index scores for the six original domains were 32.60 for open surgery, 30.32 for minimally invasive surgery, and 40.82 for robotic-assisted surgery. While the literature indicates that scores exceeding 50 are potentially detrimental to individuals, it is notable that scores for all procedure types in this study remained below this threshold. Nevertheless, robotic-assisted surgery exhibited consistently higher scores relative to both open surgery and minimally invasive surgery, indicating a greater perceived workload for robotic-assisted procedures.

Regarding the differences between scores for the 3 different phases of the surgery, a significant difference was observed in the mental demand domain, with phase 1 (patient entry to first incision) scoring higher than phase 2 (First incision to closing) (p=0.033), indicating that nurses found phase 2 more mentally demanding than phase 1 (see Fig. 4b). Descriptive statistics and effect size are provided in Table 3.

**Table 3:** SURG-TLX mental demand scores per surgical phase (summed across surgery types).

Surgical phase	Mean (SD)	Significant differences	Effect size (partial η²)
Phase 1 (Entry to Incision)	16.42 (10.53)	Phase 1> Phase 2(p = 0.033)	∼0.07 (moderate)
Phase 2 (Incision to Closure)	22.68 (10.86)		
Phase 3 (Closure to Patient Exit)	18.42 (12.16)		

### 3.2 Factors influencing workload and job satisfaction

Using inductive categorization, the factors identified by the 28 participants in question 10 and 11 of the questionnaire were grouped into seven main categories affecting workload and job satisfaction. The development of these categories, as well as the interpretation of their content, was guided by insights from the interview data to ensure alignment with the lived experiences and language of intra-operative nurses. These categories and their corresponding subcategories are detailed in this section and illustrated with quotes from the interviews. Supplementary Fig. 2, Appendix D, illustrates the frequency with which nurses mentioned the factors.

#### 3.2.1 Team dynamics

The dynamics within the surgical team play a central role in shaping both workload and job satisfaction. Nurses consistently emphasized that the skill level and demeanor of their colleagues influenced their experience. Skilled and pleasant colleagues could ease workload and contribute positively to the work atmosphere, while less experienced or incompatible team members often increased stress and complexity.

A recurring theme was the influence of the surgeon’s personality on the overall team atmosphere. Surgeons who communicated well and maintained a supportive tone helped foster a positive working environment, especially in high-pressure settings. Teamwork emerged as a critical factor: when collaboration was smooth and roles were clearly coordinated, nurses experienced lower workload and greater job satisfaction. One nurse noted, *“It makes a big difference if, at the end of the day, everyone is thanked for their hard work as a team.” (Female, 29).*

#### 3.2.2 Procedural characteristics

The type of surgical procedure itself also shaped nurses’ experiences. Participants expressed a preference for open abdominal surgeries over miminally invasive or robotic-assisted procedures. Open surgeries were often described as more engaging and dynamic, offering greater opportunities for nurses to think along and remain active throughout the procedure. As one nurse put it, *“Open abdominal surgeries are much more enjoyable than robot and laparoscopic surgeries. They are more challenging for us because you can think along, stay busy, and keep moving.”(Female, 56).*

Robotic-assisted procedures, in contrast, were sometimes perceived as monotonous or passive, particularly when the nurse's tasks were limited. Long procedures with little direct involvement could increase perceived workload despite fewer physical demands. Visibility and engagement were particularly low during certain robotic phases, leading to reduced job satisfaction.

The smoothness of a procedure—how well it flowed without interruptions or confusion—was also key. Chaotic or poorly managed procedures increased mental demands and stress. Finally, variety played a dual role: while variation in tasks and cases could increase satisfaction, rotating across different specialties or shifting between robotic and minimally invasive surgeries in a single day often elevated workload due to the preparation and cognitive switching required. One nurse noted, *“If we have to switch between different types of surgeries like laparoscopic, robotic-assisted surgery, and then laparoscopic again, it becomes very inconvenient. Each change requires extra preparation, elevating workload.”(Female, 32).*

#### 3.2.3 Preparation and equipment

Preparation was a consistent determinant of both workload and satisfaction. Nurses highlighted that when setups were incomplete or plans unclear, they had to compensate under pressure, increasing their workload and reducing satisfaction. Robotic-assisted surgeries, in particular, required more extensive preparation. One nurse explained, *“The preparation for robotic-assisted surgery procedures is more than for an open or laparoscopic procedure.”(Female, 45).* Technology-related challenges—especially with robotic systems—further added to workload. Equipment malfunctions or delays in system readiness were frequent stressors and led to feelings of frustration and inefficiency. These issues contributed to lower satisfaction and, in some cases, made nurses feel disconnected from the surgical process.

#### 3.2.4 Working environment

Physical elements of the operating room environment, such as lighting, sound, and space, also impacted nurse experiences. Dim lighting, often used in minimally invasive and robotic surgeries, was noted to increase strain and reduce comfort. While background music could create a relaxed atmosphere, excessive or disruptive noise elevated stress levels. Crowding in the operating room—whether from additional personnel, observers, or equipment—was another challenge. This was especially prevalent in robotic-assisted surgeries, where large consoles and robot arms took up significant space, reducing nurses' freedom to move and increasing physical and mental workload. As one nurse stated: “*It does feel crowded with the robot, and because you don’t have much to do you just sit down.”* (Female, 29).

#### 3.2.5 Organizational factors

Beyond the immediate operating room setting, broader organizational factors also influenced nurses' workload and job satisfaction. Poor surgery scheduling, including back-to-back complex procedures or inadequate breaks, led to fatigue and reduced morale. High work pressure, often stemming from staff shortages or urgent cases, compounded these issues. Some participants also cited salary disparities as a demotivating factor, with several colleagues leaving for better-paying agency jobs: *"I get that you would do that. A colleague of mine is a single mother with two kids, she can afford to buy a house if she starts working as a contractor, but not if she stays working here."(Female, 56).*

The ability to take breaks during procedures—particularly during long robotic cases—was another point of concern. Nurses reported that being unable to step out of the operating room, even briefly, negatively impacted both workload and job satisfaction.

#### 3.2.6 Appreciation and recognition

Feelings of appreciation played a major role in shaping job satisfaction. Nurses described how simple gestures of recognition could have a significant impact on their morale. As one participant explained*, “A simple ‘Hey guys, we worked really hard together today, thank you so much’ can have a large impact.”* (Female, 55). Conversely, a lack of appreciation—especially when coupled with dismissive comments from surgeons—could deeply affect motivation and self-worth. One nurse recalled, *“A specialist once commented on my work: ‘Even a monkey could learn to do that.’” (Female, 32).* Nurses also reported higher satisfaction when they were actively involved and their contributions were valued. This sense of personal satisfaction was especially present during open surgeries, where their engagement in the procedure was often more hands-on and continuous.

#### 3.2.7 Physical demand

Finally, physical strain played a role in how nurses experienced different surgeries. Open procedures typically involved prolonged standing, which could be tiring. However, many participants reported that the engaging nature of these procedures made the physical demands more manageable. As one nurse noted, *“Open surgery generally means standing longer, but you hardly notice it because the work is so engaging.” (Female, 56).* In contrast, robotic-assisted surgeries often involved sitting for long periods, which some nurses found physically easier but mentally more draining. Heavy lifting of robotic equipment or positioning the robotic arms also added to the physical workload, particularly during setup and takedown phases.

By identifying these themes and their associated factors, we gain insight into what impacts the workload and job satisfaction of operating room nurses. To understand how frequently these factors occur—and thereby assess the urgency of the challenges they represent—we analyzed hospital data and video recordings.

### 3.3 Video analysis

The results of the video analysis encompassed a total of 4 open surgeries with a cumulative duration of 16 hours and 32 minutes, 24 minimally invasive surgery totaling 53 hours and 37 minutes, and 6 robotic-assisted surgery with a total duration of 17 hours and 30 minutes. One procedure was excluded due to complications.

#### 3.3.1 Results manual annotations

**Fig. 5:**
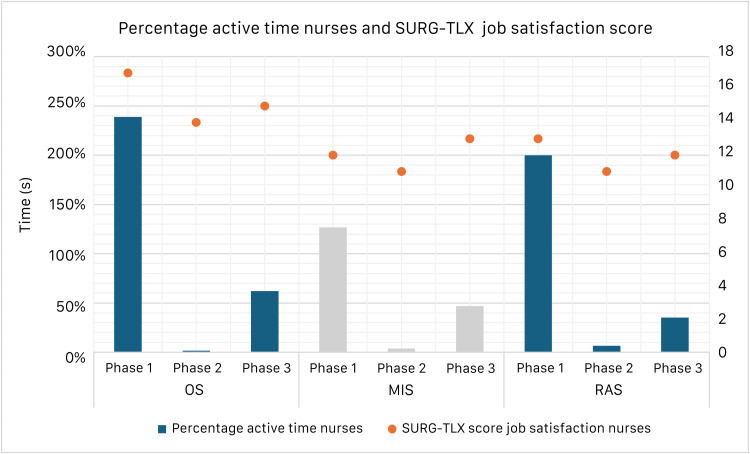
Percentage of nurses' activity time during each surgery phase, expressed relative to the average total duration of the phase and corresponding SURG TLX job satisfaction scores. Since the percentage reflects the combined activity of all active nurses, values may exceed 100%. Phase 1 includes the induction of anesthesia and surgical preparations, Phase 2 corresponds to the cutting phase, and Phase 3 corresponds to the emergence from anesthesia. The left axis represents time in seconds, while the right axis represents the SURG TLX job satisfaction score scale. Open Surgery = OS, Minimally Invasive Surgery = MIS, Robotic-Assisted Surgery = RAS.

Fig. 5 shows the percentage of nurses' activity time during each surgery phase, expressed relative to the average total duration of the phase (with the induction of anesthesia and surgical preparations combined into phase 1 of the Surgery Task Load Index, phase 2 corresponding with the cutting phase and phase 3 corresponding with the emergence from anesthesia) and the Surgery Task Load Index scores for job satisfaction. For open surgery and robotic-assisted surgery, the highest measured activity levels align with higher job satisfaction scores in the Surgery Task Load Index. Similarly, minimally invasive surgery follows this pattern in phases 1 and 2. However, in phase 3, minimally invasive surgery shows higher job satisfaction despite lower activity levels compared to phase 1, indicating a deviation from the observed relationship in other phases.

#### 3.3.2 Results automated pose tracking

Supplementary Fig. 3, Appendix E, presents the percentage of measured movement for each procedure type. This percentage represents the proportion of movement relative to the total duration of the procedure. To enable comparison between procedures of varying lengths, we calculated these percentages. Additionally, to illustrate the variation in the lengths of the 34 procedures, the x-axis of the figure displays the surgery duration. Overall, the values for all procedure types remain low, with movement rarely exceeding 10%, indicating minimal movement during procedures. A distinct difference emerges between robotic-assisted surgery and the other two procedure types, with robotic-assisted surgery showing lower movement percentages. In contrast, minimally invasive surgery displays more variability in movement patterns among these procedures. Still, it is important to note that minimally invasive surgery also includes considerably more data points than robotic-assisted surgery and open surgery. Also, open surgery had one notably longer surgery compared to the other procedures, which may have influenced the data.

**Fig. 6:**
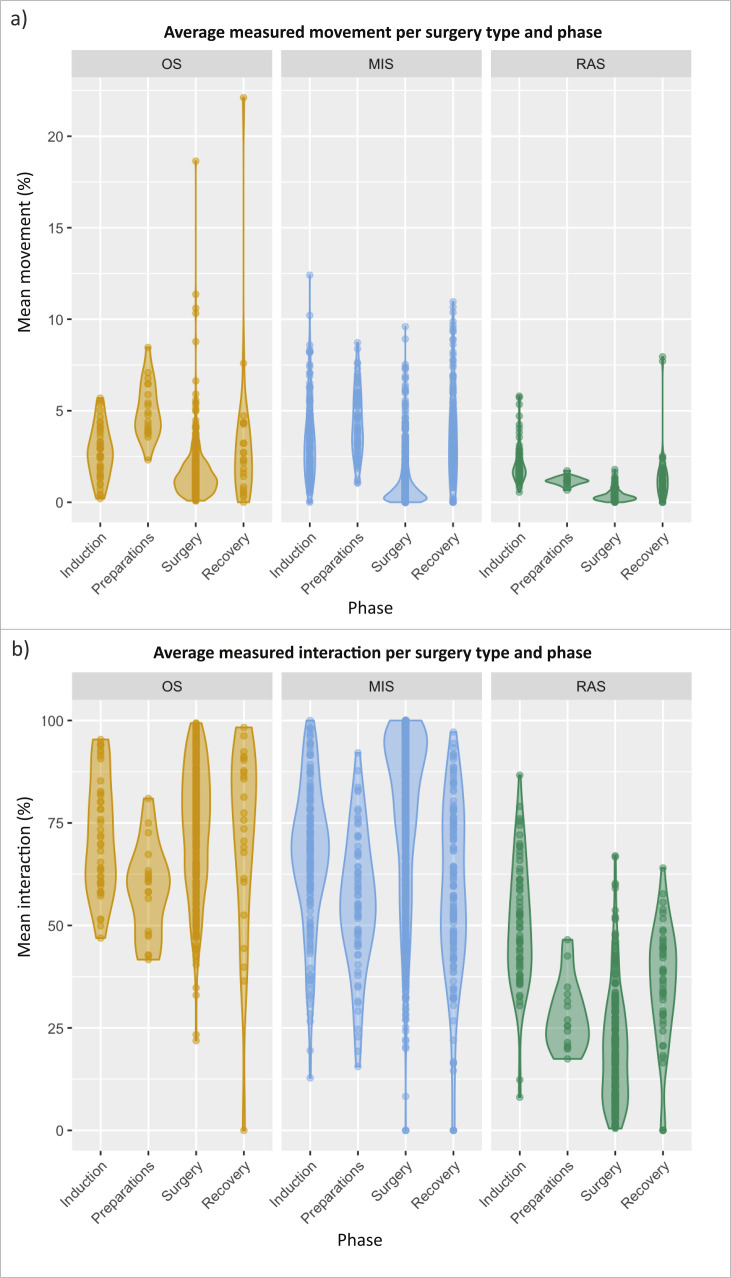
Average Measured Movement and interaction per Surgery Phase by Procedure Type. a) Illustrating the percentage of average duration attributed to measured movement during open surgery (OS), minimally invasive surgery (MIS) and robotic-assisted surgery (RAS) across different surgical phases. b) Illustrating the percentage of average duration attributed to measured interaction in OS, MIS and RAS across different surgical phases.

Fig. 6a presents the average movement percentage calculated for each surgery phase. The surgery phases were extracted through manual annotation. Overall, movement percentages are higher during the second phase compared to other phases. Fig. 6b displays the average interaction percentages in the surgery table area per surgical phase for each surgery type, where interaction refers to the number of people actively engaged around the surgical table area. In robotic-assisted surgery, interaction decreases during the surgery phase and remains lower across all phases compared to minimally invasive surgery and open surgery.

### 3.4 Hospital Data

Supplementary Table 2, Appendix F, presents a summary of metrics related to the gynecological procedures performed in 2023. The data includes total procedure duration, deviations from planned durations, patient risk scores of patients, and the time spent in the dark—defined as the periods during which the dim lights are turned on, primarily occurring during the cutting phase (phase 2) of the surgery.

Minimally invasive surgery procedures had the shortest operating times, while robotic-assisted surgery and open procedures had similar durations. During Phase 2 of the procedure (from first incision to closing), which involves dim lighting, robotic-assisted surgery required more time compared to minimally invasive surgery, a factor nurses indicated negatively affects job satisfaction, linking to questionnaire outcomes.

Open procedures often finished ahead of schedule, minimally invasive procedures stayed closer to planned durations, and robotic-assisted procedures tended to run longer, with the cumulative effect of small delays becoming significant over multiple surgeries. Patients undergoing open procedures generally had higher patient risk scores, indicating greater preoperative risk, while robotic-assisted procedures were performed on healthier patients, with minimally invasive surgery falling in between. Additionally, open and robotic-assisted procedures typically required more personnel in the operating room compared to minimally invasive surgery.

## 4 Discussion

This research employed a multi-method approach—questionnaires, interviews, hospital data and video recording analysis—to evaluate the relation between operating room technology use and intra-operative nurses' workload and job satisfaction in the Netherlands. Our study identified several key influencing factors among which are team dynamics, procedural characteristics, preparation and equipment, work environment, organizational support, appreciation, and physical demands.

In this study, we extended the original Surgery Task Load Index by adding a seventh aspect: job satisfaction. To our knowledge, this is the first time job satisfaction has been integrated into the Surgery Task Load Index framework, marking an exploratory adaptation of the tool. By measuring job satisfaction on the same 20-point scale, we aimed to gain insights into how nurses experience satisfaction during different types of surgeries and how this relates to workload. As this is a first construct, the findings related to job satisfaction should be considered exploratory. They offer a valuable starting point for further investigation but should not be interpreted as conclusive statistical outcomes.

### 4.1 Open surgeries

Intra-operative nurses consistently rated open procedures as the most satisfying, correlating with higher job satisfaction scores on the Surgery Task Load Index. Despite their longer duration, nurses reported staying actively engaged, contributing to a manageable workload. One nurse explained that the continuous involvement in open surgeries enhances satisfaction. At Leiden Univeristy Medical Center, open procedures, often involving higher-risk (patient risk score) and emergency patients, were preferred for their challenges and hands-on engagement. Additionally, open surgeries typically end sooner than planned, reducing overruns and associated stress. The simpler equipment and well-lit environment in open surgeries further ease workload and stress (Sonoda et al., 2018). Video data supports these findings, showing higher Interaction with Operating Table scores, suggesting greater team engagement.

### 4.2 Minimally invasive surgeries

Minimally invasive procedures generally involve a lower workload than robotic-assisted procedures but also yield lower job satisfaction than open surgeries. The reduced workload is likely due to the higher frequency of minimally invasive surgery cases, allowing nurses to gain familiarity and efficiency. Shorter procedure times and fewer people in the operating room contribute to a more favorable working environment. Video data also shows higher Movement and Interaction with Operating Table scores than robotic-assisted surgery, suggesting more active involvement by team members. However, job satisfaction scores for minimally invasive surgery remain lower than for open procedures. This may be due to the typically lower patient risk and emergency scores, which suggest less complex cases, reducing engagement opportunities. Nurses reported feeling less involved due to limited visibility of the surgical site and noted that dim lighting, while less frequent than in robotic-assisted surgery, still impacted satisfaction.

### 4.3 Robotic-assisted surgeries

Robot-Assisted procedures place a higher workload on intra-operative nurses than open and minimally invasive surgery and are associated with lower job satisfaction compared to open surgeries. The preparation phase of robotic-assisted procedures is particularly demanding, in contrast with the other procedures. Interviews revealed that nurses face significant stress preparing complex instruments and managing unexpected issues, aligning with previous findings (Sonoda et al., 2018). In the interviews, nurses report a lack of sufficient technical knowledge for troubleshooting, which increases their workload. Additionally, the lower frequency of robotic-assisted surgery cases means nurses have less hands-on experience, compounding stress and workload.

Surgery Task Load Index assessments show robotic-assisted procedures impose greater temporal demands than other surgeries, with actual durations often exceeding estimates. This discrepancy adds to nurse workload. The robotic-assisted surgery work environment, characterized by dim lighting, elevated noise from extra equipment, and a crowded operating room, contributes to increased distraction scores and lower satisfaction. More personnel are typically present, increasing noise and reducing space, while robotic-assisted procedures attract additional observers, further contributing to distractions.

Lower job satisfaction in robotic-assisted surgery cases may be partly due to reduced engagement; nurses report limited visibility of the surgical site and fewer tasks, especially in phase 2 (first incision to closing). This diminished involvement can lead to under-arousal, impacting both performance and satisfaction. Additionally, robotic-assisted surgery patients typically have lower patient risk scores, presenting less complexity and fewer challenges, which may also lower engagement and satisfaction. Video data supports these insights, showing that robotic-assisted procedures have lower Movement and Interaction with Operating Table scores compared to open surgeries and minimally invasive surgery, reflecting reduced activity and involvement. However, during the cutting phase, video analysis shows slightly higher activity levels in robotic-assisted surgery than in open surgery and minimally invasive surgery. This contrasts with findings suggesting fewer tasks for nurses during the cutting phase in robotic-assisted surgery. While robotic-assisted procedures generally have longer durations than minimally invasive surgery, potentially leading to longer periods of inactivity for nurses, the average durations of open surgery are comparable to those of robotic-assisted surgery. The nurses' reports of fewer tasks during robotic-assisted surgery may instead reflect the nature of these tasks, as they also described them as less engaging. It is important to note the limited number of recorded videos in this study. Larger datasets are necessary to obtain more reliable and generalizable results.

### 4.4 Reccomendations

Our findings point to several strategies to improve working conditions for intra-operative nurses. From a management perspective, scheduling should aim to reduce frequent transitions between surgical setups while still offering variety in tasks. Grouping similar procedures and allowing sufficient time between cases may help lower setup-related workload and increase engagement. From an engineering perspective, the physical presence of robotic systems often disrupts nurses’ workflows. Future designs should prioritize compact solutions, such as ceiling-mounted systems, to reduce floor clutter. Additionally, systems that function under normal lighting conditions could improve visibility and comfort for the surgical team. Finally, reduced nurse engagement during robotic-assisted procedures remains a concern. Ensuring nurses have a clear, active role in these workflows—through design or team protocols—will be key to maintaining job satisfaction.

### 4.5 Limitations

This study was conducted at Leiden Univeristy Medical Center, a university hospital with medical trainees who may take on tasks usually performed by intra-operative nurses, potentially biasing results. The study does not differentiate between scrub and circulating nurses, who share roles at Leiden Univeristy Medical Center, though research suggests these roles may perceive teamwork differently (Sonoda et al., 2018). Circulating nurses, who bear more physical demands (e.g., fetching equipment), reported higher frustration levels.

Focused on gynecological procedures, the study did not distinguish workload variations across specializations, though atmosphere and demands vary (Catchpole et al., 2016). In Leiden Univeristy Medical Center’s gynecology department, nurses aren’t expected to change robotic arms, unlike in urology, potentially reducing involvement during robotic-assisted procedures. Analysis of specialization differences was limited by nurses frequently working across departments and selecting multiple specializations in questionnaires. Physiological workload measures (e.g., electromyography (EMG), electroencephalography (EEG)) weren’t included, so findings rely on subjective assessments and video analysis, which may limit workload evaluation comprehensiveness. Workload and job satisfaction are subjective, varying by factors such as age and hierarchy tolerance.

A small sample size limits statistical reliability, as illustrated by variability in workload perceptions across domains. Five interviews were conducted, reducing generalizability and limiting the interpretive scope. With no statistically significant domain differences, Surgery Task Load Index results were left unweighted, potentially limiting sensitivity (Olthof et al., 2018). Another potential limitation of this study is the modification of the Surgery Task Load Index questionnaire by adding an additional domain: job satisfaction. While this adjustment was made to capture an important aspect of the participants' experiences that is not addressed in the original tool, it deviates from the validated structure of the Surgery Task Load Index. This could potentially impact the comparability of our findings with previous studies using the unmodified version. Future research should consider validating the modified tool or employing complementary methods to assess job satisfaction independently.

Procedure classification was manual, with challenges from varying data formats and ambiguous cases (e.g., "research under anesthesia"). Vaginal procedures (e.g., hysteroscopies) were grouped with laparoscopies, though further separation may be warranted. Inconsistent data across procedures may also introduce bias, especially in emergency classifications and personnel records.

Additional observers during robotic-assisted procedures reportedly increased workload perception due to added vigilance needs. Observer data was absent, preventing analysis of how their presence impacts workload. Patient factors beyond patient risk and emergency classifications, such as BMI, were unavailable, though they may influence workload; one nurse noted that robotic arms struggle with obese patients. Dim lighting in phase 2 (first incision to closing) of robotic-assisted surgery and minimally invasive surgery potentially confounded responses, as nurses noted increased workload in low-light settings.

We explored the potential of automated video analysis to extract objective measurements of nurse activities. Positively, these measurements aligned closely with manual annotations, demonstrating the promise of this approach for reliably tracking nurse activities. However, the algorithm metrics primarily captured lower body movements, missing finer actions such as handovers, which are crucial for understanding workload and skill demands (Quinn et al., 2023). Additionally, the video analysis algorithm encountered challenges, including obstructions, dim lighting, and an inability to differentiate procedural phases, which limited its capacity to provide detailed insights into specific workload demands. The limited variety of recorded procedures (4 open, 6 robotic-assisted surgery, 25 minimally invasive surgery) further introduced potential biases, requiring cautious interpretation of the results. These findings highlight the need for further refinement of the algorithm to capture a broader range of activities and address contextual challenges.

### 4.6 Future research

This study highlights how surgical modality influences intra-operative nurses' workload and job satisfaction, but further research is needed to unpack the underlying factors contributing to these differences. Future studies should investigate how elements such as procedural complexity, perceived stress, and quality of treatment influence workload and satisfaction across different types of surgery. In particular, real-time measures of stress (e.g., physiological indicators or observational stress markers) could provide deeper insight into how nurses experience various surgical environments.

Moreover, while this study emphasizes the importance of creating supportive operating room environments, future research should explore concrete strategies to achieve this. This includes evaluating the effectiveness of targeted interventions such as team-based communication training, stress management workshops, and role-specific technical training programs tailored to different surgical technologies. Additionally, ergonomic improvements—such as adaptable operating room layouts, more intuitive equipment interfaces, or support tools to reduce physical strain—should be systematically tested for their impact on both nurse well-being and workflow efficiency.

Investigating the interplay between organizational practices, technology use, and human factors will be essential to developing sustainable improvements in surgical care. Longitudinal studies or intervention-based research could help determine how such changes influence nurse retention, performance, and ultimately, patient safety.

## 5 Conclusions

This study used a multi-method approach to assess the workload and job satisfaction of intra-operative nurses at Leiden Univeristy Medical Center. Key factors influencing workload and job satisfaction included team dynamics, procedural characteristics, preparation and equipment, working environment, organizational factors, recognition and appreciation, and physical demands.

Generally, nurses found their workload acceptable and were satisfied with their jobs. Open procedures led to the highest job satisfaction due to continuous engagement and a manageable workload. Conversely, minimally invasive procedures, while less demanding, resulted in lower job satisfaction due to reduced involvement, simpler cases, higher frequency, and shorter durations. Robotic-assisted procedures were associated with increased workload and decreased job satisfaction, mainly due to preparation requirements, technological challenges, diminished involvement, and a less pleasant work environment.

These findings suggest that job satisfaction among intra-operative nurses is not solely determined by workload intensity but also by the level of engagement and perceived contribution to patient care. Minimally invasive and robotic-assisted procedures, despite their technological advancements, may unintentionally reduce nurses' sense of professional fulfillment. This underscores the importance of optimizing workflow and team dynamics in these procedures to enhance job satisfaction and maintain high-quality patient care.

## CRediT authorship contribution statement

**Anne M. Schouten:** Writing – original draft, Methodology, Investigation, Data curation. **Rick M. Butler:** Writing – review & editing, Visualization, Validation, Software, Formal analysis. **Carlijn E. Vrins:** Investigation, Formal analysis, Data curation. **Steven M. Flipse:** Writing – review & editing, Supervision. **Frank Willem Jansen:** Supervision. **Anne C. van der Eijk:** Writing – review & editing, Supervision. **John J. van den Dobbelsteen:** Writing – review & editing, Supervision, Methodology, Funding acquisition.

## Declaration of competing interest

The authors have no competing interests to declare.

## Appendix Supplementary materials

Image, application 1
