# Supplementary material for: Impact of operating room technology on intra-operative nurses' workload and job satisfaction: An observational study
Source: Int J Nurs Stud Adv. 2025 Apr 29;8:100341. doi: 10.1016/j.ijnsa.2025.100341 (PMC12104634; doi:10.1016/j.ijnsa.2025.100341)
Supplement: Supplementary file 1 [file mmc1.docx]

**Appendix A: Supplementary Figure 1**


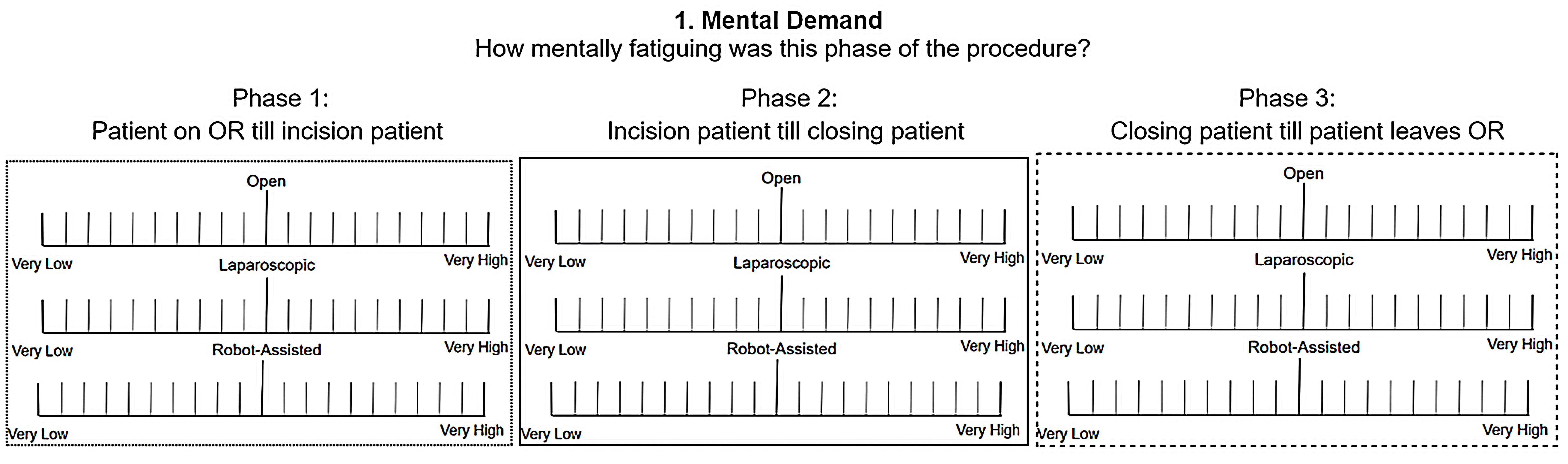


Supplementary Figure 1 SURG-TLX adaptation in which the participant scores open surgery, minimally invasive surgery and robotic-assisted surgery for each aspect of the standard SURG-TLX. Each aspect is scored this way for the three phases of the surgery: (1) entrance patient – first incision; (2) first incision – closing patient; (3) closing patient – exit patient.

**Appendix B: Data management plan**

| Name and contact details | *Ir. A.M. Schouten*  [*A.M.schouten@tudelft.nl*](mailto:A.M.schouten@tudelft.nl) |
| --- | --- |
| ORCID ID | - |
| Department | Gynaecology |
| Supervisor(s) | - Prof. Dr. F.W. Jansen, LUMC, Head of Department of Gynaecology - Dr. ir. J. Dijkstra, LUMC, Department of Radiology (Div. of Image Processing) - Dr. J.J. van den Dobbelsteen, TU Delft, BioMechanical Engineering |
| Project title | FLOW: Fresh Look at Operating room workFlow |
| Project background | Many studies have highlighted the importance of OR efficiency optimization. As a consequence, OR’s have changed a lot in the last decades. Reassessing the workflow within the complex processes of the OR is therefore valuable. Obtaining reliable estimations of the duration of procedures and communicating the progress of interventions in real-time have been identified as crucial for the improvement of the efficiency of the OR. By doing so, the medical staff can anticipate and better manage the workflow. This improves the efficiency both during a procedure as well as the sequence between different procedures. Furthermore, automated recognition of OR procedures could lessen the administrative burden of the medical staff. |
| Description of your research | The objective of this study descriptive. It has the aim of providing insights in the daily workflow of an OR. Based on these insights, technical support with new methods for understanding, real-time monitoring and management of workflow can be generated. This way, efficiency and patient safety could be increased in the OR environment. A set of operations will be filmed on the OR of the gynaecology department. This study is non-comparative, non-interventional, single-site and observational. There is no formation of study groups who will be compared. |
| Project duration | Start: *01/05/2021*  End: *01/05/2022* |
| Name and contact details data management expert LUMC*^[[1]](#footnote-1)^* | Petra van Overveld  [P.G.M.van_Overveld@lumc.nl](mailto:P.G.M.van_Overveld@lumc.nl)  071-526 3140 |
| Funding body(ies) | Not applicable |
| Grant number | Not applicable |
| Partner organisations | Technical University Delft, Medical Delta |

About this Data Management Plan

| Date written | 30/03/2021 |
| --- | --- |
| Date last update | 30/03/2021 |
| Version | 1.0 |

**Changes to earlier versions of this Data Management Plan**

| **Component** | **Progress / Execution** |
| --- | --- |
| 1. Data collection |  |
| 2. Data documentation |  |
| 3. Data storage and security |  |
| 4. Data access, sharing and reuse |  |
| 5. Data preservation and archiving |  |
| 6. Additional information |  |

| **1. Data collection**  Describing the data you will be creating/collecting | |
| --- | --- |
| 1.1 | **How will you collect, create and/or capture your data?** |
|  | A set of GoPro video cameras will be mounted to the walls in OR9 with suction cups. The GoPro cameras will run on batteries. After each procedure, the battery will need to be swapped. At the same time, the memory cards can be taken out to transfer the data to a hard-drive using a computer. New batteries and memory cards will be placed in the cameras, to save time. During the next surgery the first set of batteries will be charged and the data from the memory cards will be transferred and deleted. |
| 1.2 | **What is the format and estimated size of the data?** |
|  | \| Data stage \| Specification of dataset \| Software choice \| File format \| Data size estimate \| \| --- \| --- \| --- \| --- \| --- \| \| *Raw data* \| Video recordings + audio \| GoPro software installed on equipment \| MP4 \| 150GB \| \| *Processed data* \| Body part coordinates \| Pose Estimation \| JSON/XML \| < 1GB \| \| *Results* \| Events or activities \| x \| JSON/XML \| < 1GB \| |
| 1.3 | **Will the project use existing data?** |
|  | ☐ No  **☐ Yes**  If yes: what kind of data will you re-use?  ☐ Data collected by myself / my research group (previous research)  **☐ Care data from electronic health records (EHR)**  ☐ Data from academic collaborators (consortium partners usually with own PI)  ☐ Data from commercial collaborators (e.g. pharmaceutical company)  ☐ Data from an open access database / archive / repository  ☐ Data from a specialist commercial data provider  ☐ Data from an existing cohort, biobank or registry  ☐ Other data (please specify)  Is there an agreement for the use of existing data?  **☐ No**  ☐ Yes, I have a data transfer agreement (DTA)  ☐ Yes, this is written down in a consortium agreement  ☐ Yes, this is written down in a research agreement  ☐ Yes, other (please specify) |
|  |  |

| **2. Data documentation**  Documenting your data to help future users to understand and reuse it | |
| --- | --- |
| 2.1 | **How will files and folders be named and structured? How will versions and changes be handled?** |
|  | FLOW_videoRaw_BACKUP  FLOW_audio  FLOW_videoEdited  FLOW_coordinates  FLOW_results  FLOW_software (version control LUMC Github with access rights)  FLOW_documents |
| 2.2 | **What metadata (standard) will be used to describe the dataset?^[[2]](#footnote-2)^ (business metadata)** |
|  | **☐ Generic metadata standard (*e.g.* Dublin Core^[[3]](#footnote-3)^)**  ☐ Specialised metadata standard  ☐ Other metadata (standard)  *Please describe briefly*: |
|  | The Dublin Core metadata standard will be used to describe the total dataset, and the subsets containing clinical and questionnaire data. The metadata will be created using the Dublin Core generator. |
| 2.3 | **What metadata (standard) will be used to describe and/or standardize data and variables? (technical metadata)** |
|  | **☐ No metadata standard is used, but I will provide a detailed description of variables (dictionary)**  ☐ Generic metadata standard (*e.g.* SNOMED, ICD10; see RDA Metadata Directory^[[4]](#footnote-4)^ for examples)  ☐ Specialised metadata standard  ☐ Other metadata (standard) |
|  | The recordings will be either in image format JPEG or PNG, or it will be video format MP4.  Where possible SNOMED coding will be used for clinical variables. Data from HiX include diagnosis information using ICD10 coding. A dictionary for clinical data from HiX will be produced from variable descriptions from DIG. |
| 2.4 | **What supporting information / documentation will you create to enhance understanding of the data?** |
|  | The research protocol will be stored with the data after approval by the METC.  A data dictionary (code book) will be available for the clinical data. A readme.txt with a list of all available files and a description of their contents will be created at the end of the project, before archiving the data. Lab journal entries will be exported as pdf.  After the project has been completed, metadata documentation will be created, specifying all relevant information needed to replicate our studies. We will also include the necessary software and tools needed for reuse and state whether embargoes, licences, commercial objectives or other conditions (like stated in informed consent agreements) have been imposed on the reuse of data.  Readme.txt:  README.md file   - Will describe folder structure, containing files, file names and their format *yyyymmdd_[type]_[name]_[version]* - Camera setup and recording conditions - Type of proceduresComments in code |
| 2.5 | **Indicate which laws or permits apply to your study^[[5]](#footnote-5)^**  **☐ Algemene verordening Gegevensbescherming (AVG) / General Data Protection Regulation (GDPR)**  **☐ Code of conduct for medical research (*e.g.* GCP)**  **☐ Kwaliteitsborging mensgebonden onderzoek (Quality Assurance for Research involving Human Subjects)**  ☐ Wet Medisch Wetenschappelijk onderzoek met mensen (WMO) (Medical Research Involving Human Subjects Act)  ☐ Approval by ethical committee for human research (METC/CCMO)  **☐ Verklaring geen bezwaar from METC (letter of non-objection)**  ☐ Wet op geneeskundige behandelingsovereenkomst (Medical Treatment Contracts Act)  ☐ Gedragscode goed gebruik van lichaamsmateriaal (Code of conduct responsible use of human tissue)  **☐ Report the collection of (in)directly identifiable (research) data to the Data Protection Officer^[[6]](#footnote-6)^**  ☐ Permission for animal experiments Centrale Commissie Dierproeven (CCD)  ☐ Permission for working with genetically modified organisms (GGO)  ☐ Other (please specify)  *Please add additional information if needed:* |
|  |  |

| **3. Data storage and security**  Ensuring that all research data are stored securely and backed up or copied regularly during your research | |
| --- | --- |
| 3.1 | **Where will you store the different parts of your data?** |
|  | ☐ On departmental network storage drive (e.g. I:-drive)  ☐ On personal network storage drive (e.g. H:-drive)  **☐ On a protected network storage drive (e.g. DataSafe)**  ☐ In a safe shared Virtual Research Environment (e.g. SharePoint Office 365)  ☐ In a safe personal Virtual Research Environment (e.g. OneDrive Office 365)  ☐ Physical storage (*e.g.* USB, external hard drive)  ☐ Cloud service (*e.g.* SURFdrive, Mendeley data)  ☐ Data management system (please specify)  **☐ LUMC long-term storage**  ☐ Other (please specify) |
|  | All data will be stored on DataSafe. Jouke Dijkstra, the project supervisor, will be in charge of the data and access to the data. |
| 3.2 | **Are there any commercialisation, ethical or confidentiality restrictions about handling your data during your research?** |
|  | ☐ No  **☐ Yes** |
|  | Informed Consent needs to be signed by both personnel and patients. |
| 3.3 | **Will you be doing research involving human subjects and/or human material?**  ☐ No, I will not be doing research involving human subjects and/or material  ☐ Yes, anonymized human material  ☐ Yes, pseudonimized human material  **☐ Yes, pseudonimized human data**  ☐ Yes, pseudonimized human data and material |
|  | The entire OR will be filmed, this includes patients and employees. However, in this research the video data will be translated to spatial data to detect activities and the face of patients will be blurred. |
| 3.4 | **How will privacy be managed during the project? (if applicable)** |
|  | The combination of the distance of the video cameras, installed high up the walls to the employees and the employees wearing face and hear masks, make that all persons present in the OR are hard to recognize in the first place. In addition, the face of the patient will be blurred to ensure privacy. After recording, an algorithm will distract the spatial data of humans from these recordings and these will be used to extract the events. This further diminishes privacy issues.  The data will be stored in the DataSafe and the supervisor of the project will be in control of the data. When publishing about this research, the faces of personnel will always be blurred. |
| 3.5 | **How will access to the data be managed during the project?** |
|  | The data will be stored on the DataSafe with access rights for only a select group of people that are involved in the research project. |
| 3.6 | **Is there any non-digital data or outputs that the project will generate? How will the non-digital data be handled and stored?** |
|  | Yes, the Informed Consents of patients and employees, which are used to get permission to film, are non-digital. These will be stored in a locked closet on the department of Radiology. They will also be scanned and stored in the DataSafe folder with the key file, since they contain personal information about the patients (name). |
| 3.7 | **What costs do you expect for storage and data management during the project? How will these costs be covered?** |
|  | The expected size of the data will is estimated to be 150GB. 1TB will cost 100 euros for 1 year LTS. Hosting this data for 15 years will cost 225 euros. |

| **4. Data access, sharing and reuse**  Managing access and security, sharing your data | |
| --- | --- |
| 4.1 | **Are there any restrictions placed on sharing / reuse of some / all of your data? Will you share your data open access or with restricted access? Is there an embargo period before sharing your data?** |
|  | Because of ethical reasons and restrictions by the law, the raw video data of this project will never be shared without restrictions. Data will only be shared with people within the LUMC working on projects that follow up on this line of research. |
| 4.2 | **If data is shared with restricted access: do you have a Data Transfer Agreement (DTA) available for reuse of your data?** |
|  | No, data is only used within the LUMC. |
| 4.3 | **If intending to share any part of the data, do your consent forms and/or consortium agreement include information about intentions for sharing, retention of data and steps taken to protect participants privacy and confidentiality?** |
|  | ☐ Yes  ☐ No  **☐ Not applicable** |
| 4.4 | **Who is responsible for your data and has authority to grant (additional) access to your data?** |
|  | ☐ You  ☐ A colleague from the project  **☐ Supervisor**  ☐ Data Access Committee  ☐ Funder  ☐ Collaborator / research partner organisation  ☐ Other  *Please describe briefly how this is arranged during your study and for the long term. Specify a person or (preferably) a role:* |
|  | This study is only just being set up. The project is financed by Medical Delta within a collaboration between TU Delft and LUMC. Jouke Dijkstra, who acts as LUMC supervisor will be in charge of granting access to the data. |

| **5. Data preservation and archiving**  Preserving your data | |
| --- | --- |
| 5.1 | **Please describe which parts of your data you will select for archiving and motivate why you would not archive (parts of) your data.** |
|  | All data needs to be archived. Unedited but compressed video data needs to be used to train algorithms in future research. The PIFs are necessary to be archived by law. The research results are necessary for the understanding and as a source for future research. |
| 5.2 | **How long must your data be preserved?**  *Minimal preservation time for different types of research:  Pre-clinical research: 10 years  Clinical research: 15 years*  *Pharmaceutical clinical research: 20 years* |
|  | 15 years |
| 5.3 | **Are there any requirements regarding the disposal of data?** |
|  | yes; The head of department is responsible for approval of data disposal.  Paper informed consent forms will be disposed of in special locked confidential paper containers and will be destroyed as confidential material according to DIN66399-2 guidelines. |
| 5.4 | **How will you ensure data findability and availability for the long term?** |
|  | ☐ I will archive my data in a general data database / archive / repository (e.g. DANS/EASY)  ☐ I will archive my data in a field-specific data database / archive / repository (e.g. EGA for genetic data)  **☐ I will not archive my data outside LUMC, but will ensure long term findability and availability (specify)** |
|  | Data will be stored on a drive that is accessible to the LKEB department. Jouke Dijkstra, the project supervisor, will be in charge of the data when this research part is finished. No plans are set for the deposit of metadata in a repository as of yet. Scripts might be published on GitLab. |
| 5.5 | **If archiving in a database / archive / repository, does it provide:**  **A CoreTrustSeal ^[[7]](#footnote-7)^?**  ☐ Yes (true for for example DANS/EASY, IKNL, 4TU.ResearchData)  **☐ No**  **A Persistent Identifier (PID)?**  ☐ Yes, a DOI  ☐ Yes, a different PID  **☐ No** |
|  | Scripts might be published on GitLab. If so, we will make the work on GitLab citable by archiving the GitLab repository and assigning a DOI with a data archiving tool. |
| 5.6 | **What will you do to prepare your data for archiving? Will there be extra costs for this preparation?** |
|  | Compress data, create README file, create separate metadata set, define Informed Consent content. |
| 5.7 | **What costs (if any) will be associated with long-term storage of your data? How will these costs be covered?** |
|  | The costs for storage of about 150GB for 15 years will be 225 euros and will be covered by the Impact Project. |

| **6. Additional information** | |
| --- | --- |
| 6.1 | **Here you can put any additional information that you were not able to list in the boxes above** |
|  |  |

**Appendix C: Supplementary Table 1**

Supplementary Table 1 Annotation scheme used for the manual annotation of the videos. The results of these annotations were used to validate the outcomes retrieved from the automated analysis with AlphaPhose.

| **Surgical phase** | **Room characteristics** | **Operating Room team actions** | **Scrub nurse actions** | **View blockers** |
| --- | --- | --- | --- | --- |
| Patient in operating room | Light turned off | Number of staff members present over time | Not active | Patient covered with sterile sheets |
| Start anesthesia | Light turned on | Number of staff interacting with operating table | Handing instruments | Patient not covered with sterile sheets |
| End of induction | Door movements | Number of active staff members | Wrapping robot in plastic | Robot positioned at patient |
| Start surgical preparation |  | Number of non-active staff members | Moving instrument table/wagon | Robot in corner of the room |
| Start surgery |  |  | Unpacking instruments |  |
| End surgery |  |  | Holding items at the operating table |  |
| End anesthesia |  |  | Retrieving items from the operating table |  |
| Patient leaves operating room |  |  |  |  |

**Appendix D: Supplementary Figure 2**

Supplementary Figure 2 Frequency of factors impacting workload and job satisfaction as reported by nurses in the questionnaire, categorized into seven groups.

**Appendix E: Supplementary Figure 3**


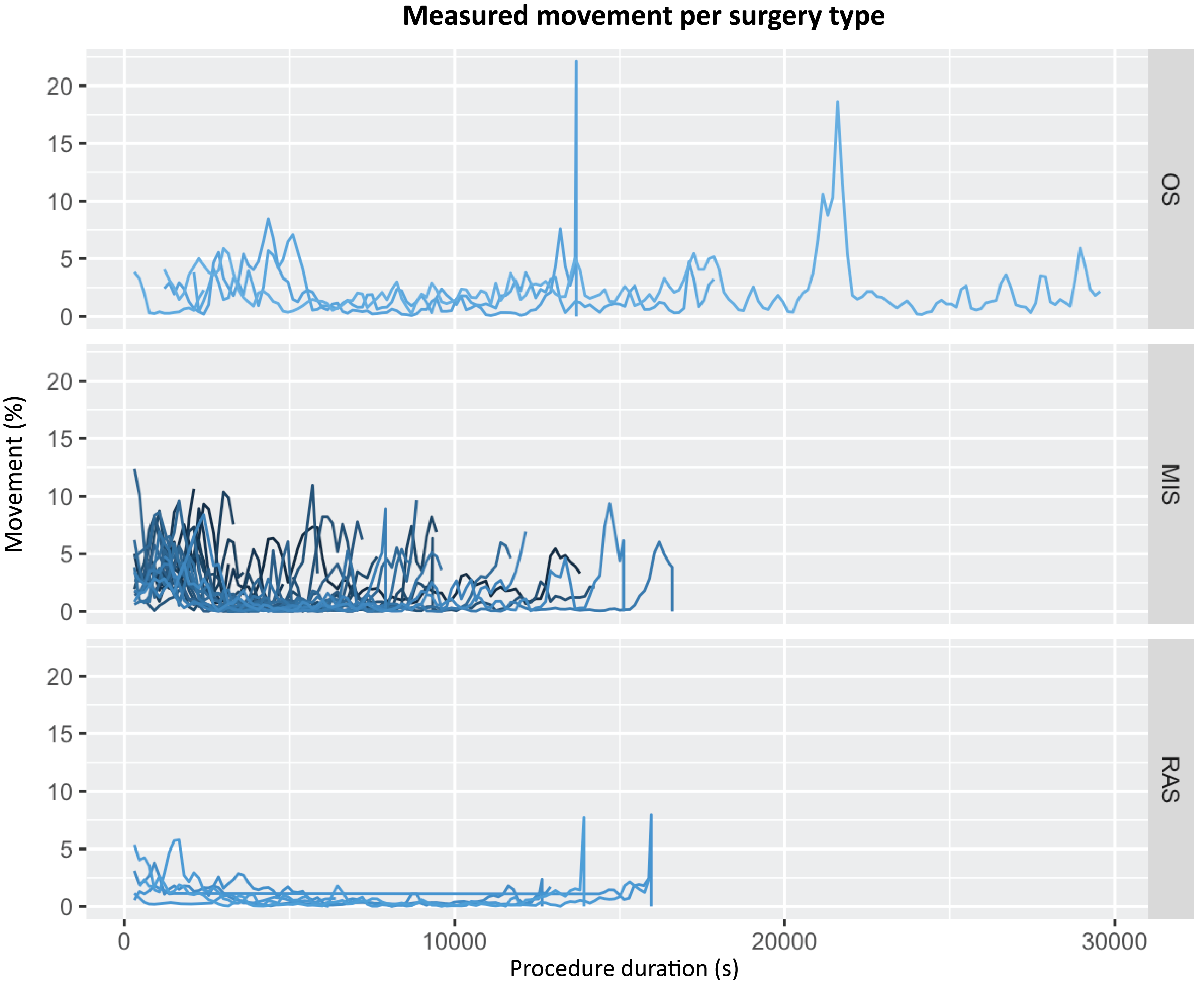


Supplementary Figure 3 Percentage of Measured Movement by Procedure Type: Illustrating the percentage of total duration attributed to measured movement for open surgery (n = 4), MIS (n = 24), and RAS (n = 6). All 24 procedures have been individually plotted for each surgery type.

**Appendix F: Supplementary Table 2**

Supplementary Table 2 Summary of Hospital Data for Gynecological Procedures in 2023: Key metrics for open surgery (OS), minimally invasive surgery (MIS), and robotic-assisted surgery (RAS), including total procedure duration, difference from planned duration, ASA scores, and time spent in the dark during the cutting phase.

| **Procedure type** | **Total duration (min)** | **Difference from planned duration (min)** | **ASA score** | **Time in the dark (min)** | **Number of people in operating room (mean)** |
| --- | --- | --- | --- | --- | --- |
| OS (N=148) | 249.32 ± 107.24 | −13.32 ± 59.09 | 2.05 ± 0.68 | - | 11.43 |
| MIS (N = 236) | 120.26 ± 65.52 | −1.66 ± 29.26 | 1.78 ± 0.61 | 71.56 ± 55.60 | 9.82 |
| RAS (N = 27) | 249.41 ± 74.61 | 6.26 ± 66.75 | 1.39 ± 0.50 | 188.58 ± 70.65 | 10.42 |

1. The [Research Support Desk](http://iprova.lumc.nl/management/hyperlinkloader.aspx?hyperlinkid=e9322414-a1c9-45a1-a00a-2c93fc84bdfd) can put you in contact with the most suited data expert if you don’t know who to contact. [↑](#footnote-ref-1)
2. See [http://www.dcc.ac.uk/resources/metadata-standards](http://iprova.lumc.nl/management/hyperlinkloader.aspx?hyperlinkid=7888234e-8c3e-444c-8962-c1fd3d180e36) or [http://en.wikipedia.or~/wikt/Metadata_standards](http://iprova.lumc.nl/management/hyperlinkloader.aspx?hyperlinkid=170e4070-7ca4-4d1c-9369-005d807595b3) for the

   relevant repository. [↑](#footnote-ref-2)
3. [https://nsteffel.github.io/dublin_core_generator/](http://iprova.lumc.nl/management/hyperlinkloader.aspx?hyperlinkid=d37ab8c2-7d66-4ff0-8510-ec89753a1687) [↑](#footnote-ref-3)
4. [http://rd-alliance.github.io/metadata-directory/standards/](http://iprova.lumc.nl/management/hyperlinkloader.aspx?hyperlinkid=f0eb08d4-ffaf-42bf-836a-346895c5ac0c) [↑](#footnote-ref-4)
5. On the CCMO website you find information that assists you in the reviewing procedure for medical/scientific research: [https://english.ccmo.nl/investigators/guide-to-the-review-procedure](http://iprova.lumc.nl/management/hyperlinkloader.aspx?hyperlinkid=e8de9866-db7b-4104-8069-6ec3015d2e5e) [↑](#footnote-ref-5)
6. [https://www.albinusnet.nl/weten-en-regelen/onderzoek/integriteit-en-privacy/meldenverzamelenonderzoeksdata/?setlanguage=English&setcountry=en](http://iprova.lumc.nl/management/hyperlinkloader.aspx?hyperlinkid=edd093e4-5dfb-4c25-9fb6-2f9b2fa61124) [↑](#footnote-ref-6)
7. If not sure, check [https://www.coretrustseal.org/why-certification/certified-repositories/](http://iprova.lumc.nl/management/hyperlinkloader.aspx?hyperlinkid=53f0f330-5624-4a20-ac90-029c3a550197) [↑](#footnote-ref-7)
